# Editorial: Chemotherapy and other pharmacotherapies for canine neurological disorders

**DOI:** 10.3389/fvets.2023.1323496

**Published:** 2023-10-30

**Authors:** R. Timothy Bentley, Timothy M. Fan, Mark Lowrie

**Affiliations:** ^1^Department of Veterinary Clinical Sciences, Purdue University, West Lafayette, IN, United States; ^2^Department of Small Animal Clinical Sciences, University of Liverpool, Liverpool, United Kingdom; ^3^Department of Veterinary Clinical Medicine, University of Illinois at Urbana-Champaign, Champaign, IL, United States; ^4^Neurology, Movement Referrals, Runcorn, United Kingdom

**Keywords:** brain, glioma, neoplasia, MUE, encephalitis

Chemotherapy is routinely used in canine neurology, especially for neoplastic and inflammatory syndromes. Our knowledge is generally broad but shallow: many therapies have been addressed with a single publication, but rarely is one therapy studied across multiple reports or institutions.

This Research Topic therefore focused on chemotherapy and related pharmaceuticals for two of the most common groups of central nervous system diseases in dogs. The first being neoplasia, including glioma. The second being the broad swathe of inflammatory syndromes currently referred to as Meningoencephalitis of Unknown Etiology (MUE).

Jose-Lopez analyzed over 100 canine intracranial gliomas in the literature treated with chemotherapy, the majority of which were histologically confirmed. Seventeen articles were analyzed for details of chemotherapy protocol, whether the gliomas were histologically confirmed, and survival times. Importantly, additional therapy was considered (e.g., surgery or radiation). Following this meta-analysis, the veterinary profession still requires unification for defined standards of care. For example, the standard of care for human glioblastoma is maximal safe resection followed by radiotherapy (60 Gy in 30 fractions) with concurrent temozolomide (75 mg/m^2^/day for 6 weeks, followed by six maintenance cycles of 150–200 mg/m^2^/day for 5 days, once every 28-days). The debate over optimum therapy for canine glioma continues, especially the best “bang for the buck” given owners' financial limitations. Chemotherapy as the sole therapy has poor efficacy, as does surgery; both result in outcomes comparable to palliative therapy. However, as Jose-Lopez notes, combinations of surgery or radiation therapy with chemotherapy have shown some promising results. Our literature continues to advance, with a fairly consistent theme that case series of presumed gliomas without histological confirmation have an apparent survival “boost” over histologically confirmed case series. There is well-recognized confusion on MRI between glioma and benign diseases such as cerebrovascular accidents and granulomas (1–4). It is therefore important to know both the typical outcome of histologically confirmed gliomas, *and* the typical outcome of treatment for an intra-axial lesion that is probably a glioma, but might not be.

Yanke et al. performed a vital step toward the possible introduction of benzimidazoles to glioma therapy. These safe and readily available anthelmintics have multiple anti-neoplastic actions *in vitro*. This pharmacokinetic study compared serum and CSF concentrations of mebendazole, resulting in a dose that should be efficacious; CSF concentrations exceeded the IC_50_ established by previous *in vitro* investigation. CSF concentrations have historically been used to predict brain tissue concentrations. On the one hand the normal blood-brain-barrier can be more robust than the blood-CSF-barrier, but on the other hand the blood-brain tumor-barrier is sometimes much more permeable (5).

Lyseight et al. describe the use of intrathecal chemotherapy for a case of multicentric lymphoma with involvement of the spinal cord. This route of administration, which effectively bypasses the blood-brain-barrier (including the blood-spinal cord-barrier) is underutilized in canine medicine compared to human oncology. There was a clear, although short-lived, clinical response to a single intrathecal administration (cytosine arabinoside and methotrexate, administered via the cisterna magna). All neurological deficits resolved for almost a month, as did the spinal pain. Unfortunately, the owner refused additional therapy upon relapse, but the report provides yet more evidence that intrathecal chemotherapies should be considered in dogs as in humans.

For MUE pharmacotherapy, Beasley and Shores provide detailed insights into the use of multiple therapies. Therapy for MUE remains based on a mixture of literature and clinician preference. According to the auspices of evidence-based medicine, much of our knowledge is only level 3, although arguably we have papers that rise to level 2+ or even 2++ (6). We continue to lack randomized controlled trials (RCTs), let alone level 1++ evidence (6). Until the veterinary profession routinely conducts RCTs, we must continue to work from retrospective or uncontrolled studies, supplemented with guidelines. Steroidal therapy is almost universally agreed upon for this group of diseases, but the initial dose and the speed of the taper remain to be assessed by the scientific method. Beasley and Shores use a combination of literature review and expert opinion to recommend a preliminary prednisone dose, an immunosuppressive dose to be started after infectious disease tests come back negative, and a tapering protocol. This is followed by detailed protocols for cytarabine and cyclosporine in particular.

Jeffery and Granger previously published a systematic review of 1962–2008 MUO cases (7). They return with a review of published cases since 2009. Almost 700 cases were available. One issue they discuss is the uneven exclusion of the potentially most severe cases. Studies of prednisone-only tend to include all patients started upon treatment. Studies of adjuvant medications tend to only include the patients who survive a minimum amount of time, possibly excluding the “worst doers”. Our literature has historically given the impression that steroid-only treatment is associated with a worse prognosis, but the reason for this remains unproven. This issue could be appropriately addressed by an RTC. Their 2009 review concluded that no single treatment regimen is clearly superior, and unfortunately this 2023 publication reaches a similar conclusion. In fact, the evidence for prednisone-only treatment seems comparable to the evidence for adjunctive medications. Nonetheless reams of valuable data are presented, including the various combinations of dose and route for cytarabine.

Finally, Lowrie provides a path for the future, with recommendations for an RCT for MUE. This includes the recommendation to perform an “intention-to-treat” RCT, which should overcome the inadvertent exclusion of the “worst doers” above. A hypothetical example of 100 prednisone-only dogs vs. 100 prednisone-adjuvant medication dogs is given, where both treatments result in 30 deaths. With the common “per-protocol analysis”, due to the exclusion of 15 “worst doers” who die quickly, the apparent death rate with adjuvant medications is only 15/85 or 18%. The apparent death rate in the prednisone-only group is 30/100 or 30%. As Lowrie discusses, when an “intention to treat” approach is taken, the “true” death rate of both groups is revealed to be 30/100.

## Author contributions

RB: Writing—original draft. TF: Writing—review & editing. ML: Writing—review & editing.
